# GLP-1 agonists in the treatment of chronic kidney disease in type 2 diabetes and obesity

**DOI:** 10.1172/JCI194749

**Published:** 2025-11-03

**Authors:** Mark E. Cooper, Daniël H. van Raalte

**Affiliations:** 1 Department of Diabetes, School of Translational Medicine, Monash University, Melbourne, Victoria, Australia.; 2 Diabetes Center, Department of Internal Medicine, Amsterdam University Medical Center, Amsterdam, Netherlands.

## Abstract

Glucagon-like peptide-1 (GLP-1) was initially considered to be a hormone with a predominant role in regulating glucose metabolism by inducing insulin secretion, reducing glucagon secretion, and ameliorating insulin resistance, with the last effect being largely dependent on the induction of weight loss. In more recent years, the role of this peptide beyond metabolism has progressively been explored, including its impact on kidney physiology and kidney clinical outcomes in people with obesity with or without diabetes. Indeed, despite only modest expression of the GLP-1 receptor in the kidney, the renoprotective actions of GLP-1 and its receptor agonists have become an area of intensive investigation. This Review appraises the current status of GLP-1 peptide and its receptor agonists and focuses on the preclinical as well as recent seminal clinical findings defining the kidney benefits conferred by GLP-1 receptor agonist treatment in people living with type 2 diabetes and obesity.

## Introduction

Diabetes remains the major cause of kidney failure worldwide, representing in many countries over 50% of individuals with chronic kidney disease (CKD) who require kidney replacement therapy, specifically dialysis or transplantation (1, 2). In people with type 1 diabetes, hyperglycemia seems to be the primary driver of kidney damage, and the kidney lesions observed typically show classical glomerulopathy. However, in people with type 2 diabetes (T2D), additional kidney risk factors contribute to CKD (3). Indeed, obesity, often present in people with T2D, is also a major risk factor for CKD development (4), as are associated conditions such as elevated blood pressure and dyslipidemia. This likely explains the large heterogeneity in observed lesions in kidney biopsies in the T2D population. With the increasing use of glucagon-like peptide-1 (GLP-1) receptor agonists in the management of T2D and/or obesity, the potential impact of these drugs on the kidney has become an area of intense investigation. Furthermore, while initial clinical studies focused on patients with T2D, the kidney-protective role of GLP-1 receptor agonists has now been extended to individuals without T2D.

GLP-1 is an intestinal hormone that plays a key role in glucose homeostasis. After ingestion of a meal, GLP-1 is secreted by intestinal L cells and acts on a cognate receptor within the pancreas to stimulate insulin release, while neuronal pathways also contribute to GLP-1’s insulinotropic actions. GLP-1’s actions to improve glucose metabolism are additionally driven by the reduction of hyperglucagonemia (5). GLP-1 also plays a key role in the regulation of body weight via various well-characterized mechanisms, including delayed gastric emptying and intestinal transit, and actions in the central nervous system that reduce appetite by stimulating satiety, in particular related to hedonic feeding (6, 7). The weight loss–associated improvement in whole-body insulin sensitivity contributes to the improvements in glucose metabolism associated with GLP-1 activity. Thus, drugs that mimic the action of GLP-1, known generally as GLP-1 receptor agonists, are widely used in the management of T2D and obesity, which as outlined above are both closely linked to CKD.

It remains to be determined how GLP-1 confers its kidney benefits, as the only GLP-1 receptor that has been identified has limited expression in the kidney (8). Gene expression studies, as reviewed recently (9), have identified low mRNA levels of the G protein–coupled GLP-1 receptor in numerous kidney cell populations, including endothelial cells, vascular smooth muscle cells, and glomerular epithelial cells (also known as podocytes). The major site of expression appears to be in the renin-expressing juxtaglomerular cells (8). These cells are critical regulators of glomerular perfusion and pressure and are intimately involved in a physiological process known as tubuloglomerular feedback (TGF) (10), which is detailed below.

Most antibodies against the GLP-1 receptor are not considered to be highly specific (11), and there remains substantial controversy as to the precise expression of the GLP-1 receptor at the protein level in the kidney. Nevertheless, studies using antibodies that appear to be specific for this receptor are consistent with results from gene expression studies, demonstrating staining for the GLP-1 receptor in human and primate kidney in juxtaglomerular cells (8). These findings build on an increasing body of evidence indicating that GLP-1 exerts direct effects on the kidney. In this Review, we outline the clinical evidence for the long-term kidney benefits seen with GLP-1 receptor agonists. Next, we discuss potential mechanisms involved in the renoprotective effects conferred by GLP-1 receptor agonists based on human physiological and preclinical studies. Finally, we describe the use of GLP-1 receptor agonists and more novel incretin combinations in populations beyond T2D.

## Renoprotective effects of GLP-1 receptor agonists: clinical trials

The first clinical evidence for beneficial effects of GLP-1 receptor agonists came from the cardiovascular outcome trials (CVOTs) that were conducted with these agents, where kidney endpoints were collected as secondary or exploratory endpoints. In these studies, GLP-1 receptor agonist treatment consistently reduced urinary albumin-to-creatinine ratios (UACRs) by 20%–40% and new-onset macroalbuminuria in people with T2D (12, 13). Other improvements in surrogate endpoints included a reduction in decline of estimated glomerular filtration rate (eGFR), calculated by slope analysis (14). However, these studies did not specifically include patients with kidney disease, and the numbers of hard kidney outcomes reached (i.e., kidney transplantation, initiation of maintenance dialysis, death from kidney failure, or a sustained low GFR) were too low to make definitive conclusions on the renoprotective effects of GLP-1 receptor agonists.

The first dedicated kidney outcome trial with the GLP-1 receptor agonist semaglutide was published more recently. In the FLOW trial (15), 3,533 participants with T2D and CKD (defined by an eGFR of 50–75 mL/min/1.73 m^2^ and a UACR between 300 and 5,000 mg/g, or an eGFR of 25 to <50 mL/min/1.73 m^2^ and a UACR between 100 and 5,000 mg/g) were randomized to receive 1.0 mg subcutaneous semaglutide once weekly or placebo. The primary outcome was major kidney disease events, a composite of the onset of kidney failure (dialysis, transplantation, or an eGFR of <15 mL/min/1.73 m^2^), at least a 50% reduction in the eGFR from baseline, or death from kidney-related or cardiovascular causes. The study demonstrated a clear benefit for semaglutide, with a 24% lower risk to reach the primary outcome in the semaglutide group versus the placebo group. The results were similar for the kidney-specific components of the primary outcome (21% risk reduction), which did not include a cardiovascular endpoint. In addition, the annual eGFR decline was lower for semaglutide by 1.16 mL/min/1.73 m^2^ compared with placebo (15). Notably, 95% of the study participants were using renin-angiotensin-aldosterone system (RAAS) blockers at baseline. The number of participants using sodium-glucose cotransporter-2 (SGLT2) inhibitors was low at baseline (15%), but that number increased during the trial. The effects of semaglutide were consistent in those using renin-angiotensin system (RAS) blockers and SGLT2 inhibition compared with those not on these therapies. A recent meta-analysis confirmed little heterogeneity in terms of outcomes in the FLOW trial and in the relevant CVOTs (16). These seminal findings led to the recent FDA approval of semaglutide for the treatment of kidney disease in T2D.

Importantly, in these and other clinical trials, the safety profile of GLP-1 receptor agonists was well established. People with diabetic kidney disease (DKD) experienced side effects similar to those of people with T2D and/or cardiovascular disease. The most common side effects are gastrointestinal in nature, and are mostly mitigated by gradual uptitration schedules. Hypoglycemia is observed when GLP-1 receptor agonists are combined with insulin therapy, so clinicians and patients have to be attentive to reducing insulin dosages given the weight loss induced by GLP-1 receptor agonists. Overall, between 5% and 10% of subjects stopped treatment in GLP-1 receptor agonist trials because of side effects (17).

## Modulation of kidney risk factors underlying renoprotective effects

**Figure 1:**
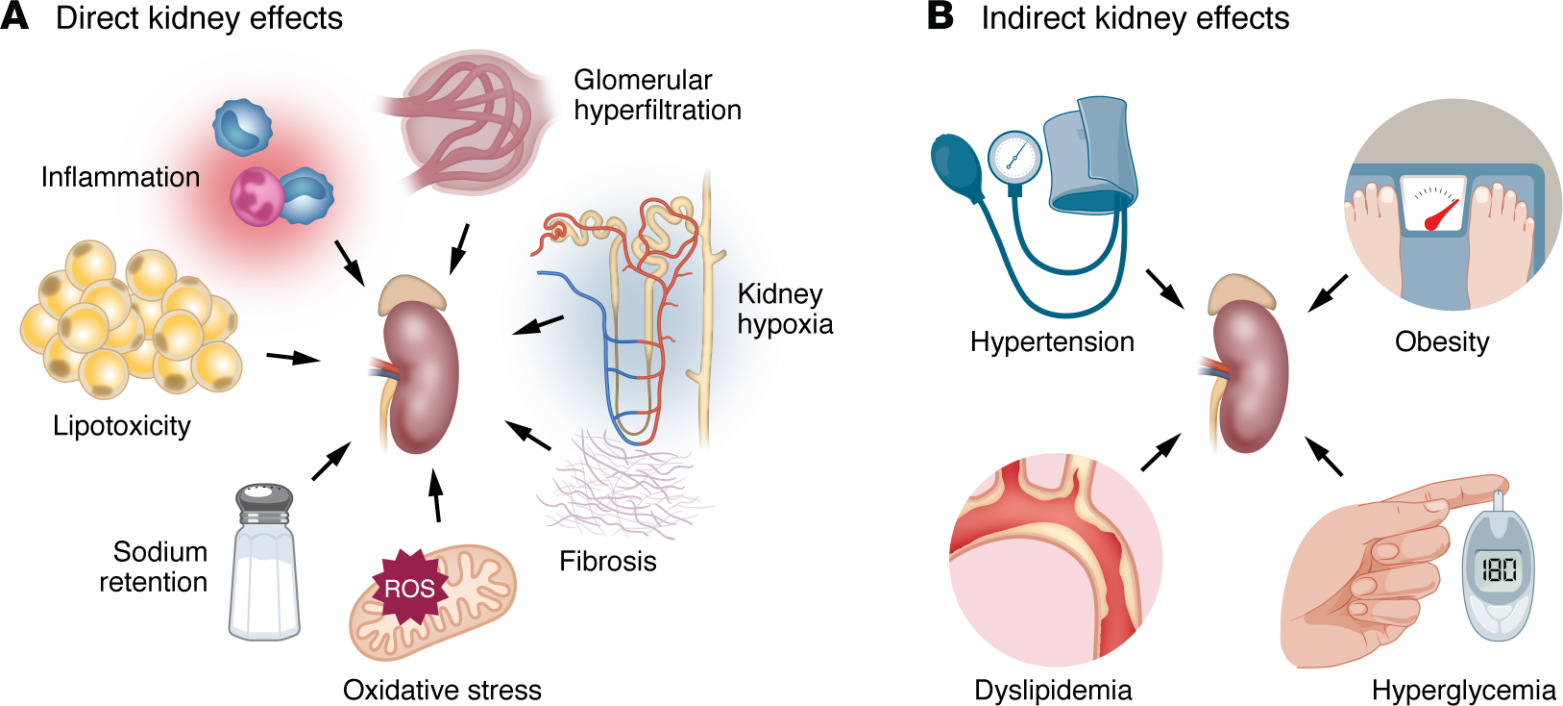
CKD drivers potentially affected by GLP-1 receptor agonist treatment. Several drivers of CKD may be impacted by GLP-1 receptor agonist treatment. Some of these effects may be related to direct kidney effects (**A**), while others may be due to effects of GLP-1 receptor agonists on kidney risk factors (**B**).

Several mechanisms have been proposed to explain how GLP-1 receptor agonists improve kidney function and outcomes (Figure 1). Some of these mechanisms may be conferred through amelioration of kidney risk factors (18). Specifically, GLP-1 receptor agonists favorably influence relevant risk factors for kidney disease, including hyperglycemia, elevated blood pressure, and increased body weight (19). Moreover, improvements in cardiac function resulting from treatment with GLP-1 receptor agonists may also contribute to the favorable kidney outcomes seen with these treatments by improving tissue perfusion (20). Thus, although much research is focused on defining the direct intrakidney effects of GLP-1 receptor agonists, it is possible that some of the benefits occur indirectly, as a result of GLP-1 receptor agonists’ ability to improve kidney risk factors, as well as via extrarenal systemic effects of these agents that are not yet fully defined, such as attenuation of low-grade systemic inflammation.

On the other hand, arguments have been raised to support direct kidney effects of GLP-1 receptor agonists. For example, the effects of GLP-1 receptor agonists on blood pressure appear to be rather modest, as recently summarized (21), and are not in line with kidney outcomes observed with other agents that provide a similar reduction in blood pressure. Similarly, regarding obesity, kidney benefits were not as clearly apparent in the lifestyle-focused Look AHEAD trial for an equivalent amount of weight loss (22). Moreover, statistical correction for changes in body weight did not attenuate the albuminuria-lowering effects of GLP-1 receptor agonists in the CVOTs (23). Finally, in the ELIXA trial, the GLP-1 receptor agonist lixisenatide was shown to reduce UACR by 39% in people with macroalbuminuria at baseline, despite very modest effects on hemoglobin A_1c_ (–0.3%) and body weight (–0.7 kg) (13). These results have stimulated a number of human physiology studies and preclinical studies that have interrogated potential direct kidney effects of GLP-1 receptor agonists using state-of-the-art phenotyping studies, which are summarized below.

## Postulated direct renoprotective effects of GLP-1 receptor agonists: human physiology studies

### Gut-kidney axis: kidney sodium handling.

Analogous to the gut-pancreas connection, which identified the incretin effect relating to insulin secretion, early studies proposed the existence of a so-called gut-kidney axis. The proposals were based on studies in humans showing that in a sodium-depleted state, an oral sodium load is more rapidly excreted by the kidneys than an intravenous sodium load (24). This rapid excretion occurred independent of changes in the levels of circulating regulators of plasma volume, including atrial natriuretic peptide (ANP) and aldosterone, and a similar phenomenon has also been suggested for the excretion of electrolytes including potassium (18). While several gut hormones secreted by intestinal enteroendocrine cells may contribute to the gut-kidney axis, studies have shown that an oral sodium load can increase endogenous GLP-1 concentrations (18). In addition, clinical studies with GLP-1 peptide or its receptor agonists have also pointed toward a potential role for this hormone in influencing sodium homeostasis. Indeed, increased urinary sodium excretion and increased urine volume induced by GLP-1 peptide and its receptor agonists have been confirmed in rodents (25), healthy volunteers (26, 27), insulin-resistant obese males (28, 29), and patients with T2D during acute infusion studies (30). These effects on urinary volume and sodium excretion could be blocked in mice using a GLP-1 receptor antagonist (31). As seen with inhibitors of SGLT2, this increase in urinary sodium excretion is only present acutely (32). The mechanisms by which GLP-1 receptor agonists induce natriuresis are unclear, but in both rodents and humans, GLP-1 receptor agonist infusion enhances kidney lithium clearance, a marker of proximal tubular sodium reabsorption, as well as urinary pH. This suggests that the effect involves inhibition of sodium-hydrogen antiporter (NHE3) (33). Other proposed mechanisms underlying GLP-1 receptor agonism’s role in facilitating natriuresis include reduction of RAAS activity through central nervous system signaling and alteration of ANP concentrations. To what extent altered kidney sodium handling is involved in the kidney-protective effects of GLP-1 receptor agonist treatment is, however, uncertain, and GLP-1 induced natriuresis is unlikely to be the major pathway underlying renoprotection.

### Glomerular hyperfiltration.

Glomerular hyperfiltration has been identified as a key factor predisposing to kidney disease. Although a generally accepted definition is lacking, glomerular hyperfiltration has been defined as a GFR above 135 mL/min/1.73 m^2^, corresponding to two standard deviations above mean GFR in healthy individuals (34). However, in people with reduced kidney mass, hyperfiltration can occur despite the whole-kidney GFR being in the normal range, a condition referred to as single-nephron hyperfiltration. Multiple factors contribute to hyperfiltration, including impaired regulation of pre- and postglomerular resistances as well as compromised tubuloglomerular feedback due to increased proximal reabsorption of sodium and chloride (34). Studies of the effects of different GLP-1 receptor agonists on glomerular hemodynamic function and hyperfiltration have yielded inconsistent results. In obese individuals, GLP-1 infusion acutely decreased creatinine clearance (29); however, in other infusion studies with the GLP-1 receptor agonist exenatide, measured GFR (mGFR) was increased in healthy volunteers (26) and unchanged in people with T2D (30). In an uncontrolled study in people with T2D, liraglutide initially lowered eGFR (35), but this result was not confirmed in placebo-controlled studies that assessed mGFR and kidney hemodynamic function by gold-standard tracer methods (36). In people with DKD, the once-weekly GLP-1 receptor agonist dulaglutide increased mGFR (37), although semaglutide appeared to initially reduce eGFR (15). Thus, in contrast to SGLT2 inhibitors that have a clear and consistent hemodynamic mode of action, it is unlikely that kidney hemodynamic actions contribute to the renoprotective effects of GLP-1 receptor agonists.

### Kidney oxygen availability.

In recent years, the interest in kidney hypoxia as a driver of DKD has been revived owing to the development of techniques that allow the study of kidney oxygen availability. The chronic kidney hypoxia theory states that in DKD, the kidneys have insufficient oxygen availability, causing tissue damage. Because of constant reabsorption, most notably of sodium, kidney tissue is second only to the heart in oxygen consumption per gram tissue. Kidney hypoxia may be driven by two mechanisms. First, it may be due to impaired oxygen delivery resulting from microvascular damage. Second, increased oxygen consumption, caused by both glomerular hyperfiltration and inefficient energy generation due to impaired insulin sensitivity, may also contribute (38).

Blood oxygen level–dependent magnetic resonance imaging (BOLD-MRI) has been put forward as a novel tool to measure tissue oxygenation. In people with CKD, lower oxygen availability was associated with more rapid kidney function decline in comparison with people with higher oxygen availability (39). Two studies have investigated the effects of GLP-1 and GLP-1 receptor agonist treatment on several aspects of kidney physiology as measured by multiparametric kidney MRI. In a study in which GLP-1 peptide was infused, GLP-1 increased both cortical and medullary perfusion (40). In contrast, prolonged treatment with the GLP-1 receptor agonist semaglutide lowered perfusion compared with placebo (41). In both studies, oxygen availability as assessed by BOLD-MRI was not changed. Thus, it remains uncertain whether alterations in kidney oxygen availability mediate the beneficial effects of GLP-1 receptor agonist treatment.

### Inflammation.

It has been well established that in both type 1 diabetes (T1D) and T2D, a chronic low-grade inflammatory state contributes to DKD, in part due to hyperglycemic insults. Both kidney cells and recruited immune cells of the innate (M1 macrophages) and the adaptive (CD4^+^ and CD8^+^ T cells) immune systems produce proinflammatory factors that damage the kidney (42). Increased levels of cytokines such as IL-6, IL-8, IL-1β, TNF-α, and IFN-γ as well as chemokines including chemokine receptor 2 (CCR2), C-C motif chemokine ligand 2 (CCL2), CCL5 (RANTES), and C-X-C motif chemokine ligand 10 (CXCL10) have been observed in kidney tissue of rodent models of diabetes, as well as in plasma and serum of people with DKD (43).

While the direct effects of GLP-1 receptor agonists on kidney inflammation are difficult to investigate in humans, in a number of studies, GLP-1 receptor agonists were shown to modulate inflammation. In peripheral blood mononuclear cells isolated from people with T2D, exendin-4 reduced the expression and production of proinflammatory cytokines and chemokines, in association with a reduction in oxidative stress (44). In people with T1D, GLP-1 infusion counteracted hypo- and hyperglycemia-induced increases in the inflammation markers IL-6 and soluble intercellular adhesion molecule-1 (sICAM-1) (45). Additional mechanisms of the effects of GLP-1 on inflammation have also been detailed in rodent models, as described below. Because human phenotyping studies have not demonstrated clear mechanisms as to how GLP-1 receptor agonists improve kidney outcomes, a reduction in kidney inflammation that is difficult to measure in humans remains a leading candidate mechanism.

### Reduction of kidney fat accumulation and lipotoxicity.

Beyond the general DKD risk conferred by obesity, fat accumulation in depots anatomically close to the kidney as well as intrakidney fat accumulation has been linked to kidney disease. Specifically, increased perirenal fat (46) and hilar fat (47) have been linked to CKD. Proposed mechanisms include physical compression of vessels and nerves, induction of glomerular hyperfiltration (48), and activation of the RAAS as well as stimulation of inflammation by local release of cytokines (49). In addition, parenchymal lipid deposition may induce lipotoxicity (50) through the formation of toxic metabolites including ceramides and diacylglyceride that hamper the function of multiple kidney cell types, including podocytes (51).

Given the effects of GLP-1 receptor agonists on body weight and fat, the question arises of whether a reduction in kidney-specific fat depots could contribute to the kidney-protective effects of GLP-1 receptor agonist treatment. Currently, a number of studies are employing multiparametric kidney MRI to assess the effects of semaglutide (NCT04865770) (52), tirzepatide (NCT05536804) (53), and retatrutide (NCT05936151) (54) on kidney fat accumulation and link these effects to changes in GFR and albuminuria. Regarding lipotoxicity, a study in kidneys of mice fed a high-fat diet (55) revealed that the GLP-1 receptor agonist dulaglutide changed the lipid content of the organ, including reductions in diacylglycerols, phosphatidic acids, phosphatidylglycerols, and triglycerides. On the other hand, levels of other lipids, including cardiolipins, which play a pivotal role in mitochondrial metabolism, were increased after dulaglutide treatment. These data indicate that GLP-1 receptor agonist–induced changes in local adipose tissue depots or parenchymal fat accumulation may contribute to their ability to protect the kidneys.

## Postulated direct renoprotective effects of GLP-1 receptor agonists: preclinical evidence

In order to define the intrakidney mode of action of GLP-1 receptor agonists, numerous preclinical studies have been performed, predominantly, but not exclusively, in models of DKD. Findings from some of these models have been difficult to interpret since in certain studies the GLP-1 receptor agonists concomitantly affected glucose homeostasis, which is itself usually associated with reduced kidney injury, independent of the mode of glucose lowering. However, one of the earliest studies, performed more than 15 years ago, administered the GLP-1 receptor agonist exendin-4 to db/db mice, a model of T2D (56). Indeed, this agonist was associated with reduced kidney injury. There were modest effects on glucose tolerance without a major effect on fasting glucose concentrations or glycated hemoglobin. These early findings provided initial critical evidence that GLP-1 analogs could potentially be renoprotective, independent of their effects on blood glucose concentrations. To further explore such a possibility, a subsequent preclinical study was performed in insulin-deficient streptozotocin (STZ) diabetic rats with exendin-4 (57). In that study, exendin-4 not only ameliorated albuminuria but conferred kidney structural benefits including reduced glomerular hypertrophy and mesangial matrix expansion in the absence of an effect on body weight, blood pressure, or circulating glucose concentrations. Concomitant effects including reduced kidney inflammation and fibrosis were also observed. This effect on macrophage infiltration has become one of the most prominent features of GLP-1 receptor agonism, as demonstrated in numerous subsequent experimental studies.

To further define how GLP-1 receptor agonists act in the kidney at a molecular level, researchers have explored various pathways implicated in kidney injury particularly in the setting of diabetes. DKD is considered to occur as a result of an interplay between metabolic and hemodynamic factors (58), with many of the putative pathways leading to injury having already been delineated (59). Therefore, what have been of particular interest are the effects of GLP-1 on these clearly defined pathways that promote kidney injury, such as oxidative stress, fibrosis via enhanced prosclerotic factor action, and inflammation.

The pro-oxidant enzyme NAD(P)H oxidase 4 (NOX4) has been shown to promote kidney injury (60), and in mice its expression can be attenuated by treatment with exendin-4 (61). This effect on renal NOX4 was further examined in STZ diabetic rats treated with liraglutide (62). In that study, GLP-1 agonist treatment led to a reduction in oxidative stress, as assessed by measurement of urinary 8-hydroxy-2-deoxyguanosine and renal dihydroethidium staining. To determine whether the reduction in oxidative stress was mediated directly by GLP-1 receptor activation, complementary in vitro studies in human mesangial cells were performed. Liraglutide inhibited NOX4 enzymatic activity, and this effect could be blocked by incubation with a protein kinase inhibitor or an adenylate cyclase inhibitor (62). Such effects are consistent with a role for the G protein–coupled GLP-1 receptor.

GLP-1 is considered antiinflammatory, but whether this represents a direct intrarenal effect or potentially a systemic action involving bone marrow sources of inflammatory cells has not been fully elucidated. A recent study demonstrated that deletion of the GLP-1 receptor using knockout technology was associated with renal injury in association with macrophage infiltration (63). Moreover, GLP-1 receptor agonism with liraglutide conferred renoprotection with concomitant effects including a reduction in the release of bone marrow progenitor cells.

How GLP-1 receptor agonists act directly on the kidney despite very low levels of expression of the only known GLP-1 receptor, which was cloned more than 30 years ago (64), remains a major question, in part due to the limited ability to accurately measure GLP-1 receptor protein expression in the kidney. Early studies suggested more widespread expression of the GLP-1 receptor on a range of cell populations within the kidney using antibodies that were subsequently considered to be nonspecific (11). Recent studies, including those carried out in the Kidney Precision Medicine Project, have shown, using single-cell RNA-Seq kidney research biopsies, the expression of the GLP-1 receptor in juxtaglomerular cells, endothelial cells, and, to a lesser extent, monocytes (https://atlas.kpmp.org/).

In CKD there is increased interest in the relative roles of local intrarenal macrophages versus infiltrating macrophages from the systemic circulation in promoting renal injury. There is also increasing evidence that these two macrophage populations produce cytokines and chemokines that can create a feed-forward cycle to accelerate intrarenal inflammation in various kidney diseases, including in diabetes (65). However, the effect of GLP-1 agonists on these macrophage subpopulations remains to be determined.

In vitro studies suggest that GLP-1 has effects on numerous renal cell populations, including glomerular endothelial cells. For example, exendin-4 inhibited the generation of the adhesion molecule ICAM-1 in these endothelial cells, and this effect could be blocked by a GLP-1 receptor antagonist (57). Similar antiinflammatory effects of GLP-1 have been reported in human macrophage cell lines (66), and systemic effects of GLP-1 agonists on macrophage infiltration have been previously reported (67). Thus, whether the antiinflammatory effects of these agents represent systemic rather than local effects on renal macrophages has not, as yet, been elucidated. Nevertheless, it is increasingly evident that the antiinflammatory actions of GLP-1 and related mimetics represent key biological effects of agonizing the GLP-1 receptor, and these findings are considered to be of direct relevance to the renoprotective effects seen with GLP-1 mimetics. Hopefully, imminent results from studies such as the REMODEL trial (52), in which detailed molecular studies are being performed in human renal biopsies from subjects who have received semaglutide, will further define the effect of these agents on macrophage biology.

Fibrosis is a key pathological hallmark of DKD, and a major role has been identified for prosclerotic growth factors such as TGF-β (68). Indeed, early preclinical studies demonstrated attenuation of renal TGF-β expression in association with reduced expression of collagens within the kidney after GLP-1 receptor agonist therapy (62). Subsequently, more sophisticated studies confirmed these findings using unbiased approaches including single-cell sequencing to define cell-specific antifibrotic effects of GLP-1 analogs in the kidney (63).

Interestingly, these beneficial effects on inflammation and fibrosis, initially identified in models of diabetes, have now been extended to non-diabetic models of renal injury including the classical subtotal nephrectomy model (63), which had been used originally to define the renoprotective role of drugs that block the RAAS (69, 70). Further studies in obese non-diabetic models have also identified renoprotection with GLP-1 receptor agonists (71), thus stimulating investigations into a potential clinical role for such agents in obesity-associated kidney disease in the absence of overt diabetes, as indicated below.

## Harnessing incretin hormones beyond GLP-1: novel incretin combinations

There is increasing interest in other incretin hormones that may act on the kidney. Some of these hormones have been targeted as part of development programs that have resulted in the generation of dual and triple agonists for the management of diabetes and/or obesity. For example, gastric inhibitory peptide (GIP), an incretin hormone discovered a decade before GLP-1 (72), has been a major target for developing newer therapies for diabetes and/or obesity. The GIP receptor is expressed in the kidney (https://atlas.kpmp.org/), although its role with respect to kidney physiology and, more importantly, in kidney diseases has been understudied. Furthermore, it remains controversial whether agonists or antagonists of the GIP receptor are the optimal approach in the management of metabolic disorders (73). With regard to the kidney, this is even less understood, but the issue may be clarified once major kidney studies of agonists such as the dual GIP/GLP-1 agonist tirzepatide are completed (discussed below). Preliminary data point toward beneficial effects of GIP agonism, as reported in findings from the SURPASS program that tirzepatide reduced UACR and slowed down eGFR declined in comparison with both placebo (53) and comparator drugs including insulin glargine (74, 75).

Another hormone that is an important target for new drugs in diabetes and obesity is glucagon. Glucagon agonism is currently being considered as a component of newer agents including as a dual agonist with GLP-1 (76) and as a triple agonist with GLP-1 and GIP (77, 78). Although the triple agonist retatrutide has been demonstrated to be a powerful agent in the setting of diabetes (78) and/or obesity (77), the kidney effects are poorly characterized. There is a larger body of literature, mostly reported over 20 years ago (79), exploring the major effects of glucagon on the kidney. Glucagon was shown to increase kidney filtration and perfusion and had profound intrarenal metabolic effects, particularly on amino acid transport and metabolism. Whether these effects are ultimately relevant to the long-term impact of such agents on the progression of kidney disease also seen in diabetes and obesity is not yet known. The importance of glucagon receptors in the kidney has been further explored in mice with kidney-specific glucagon receptor knockout (80). These mice have major kidney abnormalities including enhanced oxidative stress, enhanced activity of the inflammasome, and fibrosis, all features of CKD. This justifies the rationale for glucagon receptor agonism as part of a renoprotective strategy in diabetes and obesity. Recently, retatrutide was shown to increase eGFR and to reduce UACR (81). Cotadutide, a GLP-1 and glucagon co-agonist, reduced UACR by 50% at its highest dose of 600 μg daily in people with DKD (82).

Another approach to optimize weight loss including in diabetes involves the use of a combination of a GLP-1 receptor agonist and a long-acting analog of the β cell hormone amylin, known as cagrilintide. Although this new agent (cagrilintide/semaglutide) has not been addressed from a kidney perspective in clinical studies, it is known that amylin binds to G protein–coupled receptors in kidney and has been reported to influence proximal tubular sodium transport and activate the RAAS (83, 84). Whether these kidney actions of amylin and related molecules are relevant to DKD remains to be ascertained.

## Incretin-based therapies in obesity-related kidney disease

In recent years, studies have been expanded to investigate the effects of incretin-based therapies on kidney outcomes in overweight/obese individuals without T2D. In the SELECT trial, 2.4 mg semaglutide weekly or placebo was administered to over 16,000 patients with overweight/obesity and established cardiovascular disease (85). A prespecified secondary analysis of SELECT participants without diabetes showed that semaglutide reduced the composite kidney endpoint by 22% (86), with 75% of participants on RAAS blocker therapy at baseline. At 104 weeks of treatment, eGFR had declined less in the semaglutide arm (–0.86 mL/min/1.73 m^2^) versus the placebo arm (–1.61 mL/min/1.73 m^2^). In people with overweight/obesity and non-diabetic CKD who were included in the SMART study, 2.4 mg semaglutide once weekly versus placebo reduced UACR by 52% at 24 weeks of treatment (87). Regarding co-agonist treatment, tirzepatide was recently shown in a post hoc analysis of the SURMOUNT-1 trial to also reduce UACR in overweight/obese people without T2D (88), and similar benefits for kidney function were shown in subjects with heart failure (89). Together, these findings provide evidence for the beneficial effects of incretin-based therapies for kidney function in populations without diabetes.

## Current developments and ongoing studies

As indicated above, several studies are ongoing that aim to increase our understanding of the kidney effects of GLP-1 receptor agonists and the newer incretin mimetics (Table 1). The REMODEL trial investigates the kidney effects of subcutaneous semaglutide versus placebo in people with T2D (52). Before and after treatment, a kidney biopsy is obtained for single-cell sequencing, and multiparametric kidney MRI is performed. This study has the potential to provide strong insight into the effects of GLP-1 receptor agonism on human kidney physiology and to define potential molecular mediators of renoprotection in this context. More clinical outcome data will become available over the medium term for oral semaglutide (ASCEND PLUS trial) and the nonpeptide oral GLP-1 receptor agonist orforglipron (ACHIEVE-4) (90) (Table 1). Regarding combined incretin agents, the ongoing TREASURE study assesses the effects of the GLP-1/GIP receptor agonist tirzepatide on iohexol-measured GFR in people with CKD with or without diabetes (53). In that study, parametric kidney MRI is being conducted and kidney oxygen consumption is being assessed by ^11^C-acetate positron emission tomography. The impact of tirzepatide on clinical kidney outcomes is part of the SURPASS CVOT (T2D patients) and multimorbidity outcomes (people with overweight or obesity without T2D) (91), albeit as secondary endpoints.

Regarding the GLP-1/GIP/glucagon tri-agonist molecule retatrutide, the TRIUMPH-Outcomes program (54) will assess kidney outcomes as a co-primary endpoint, while mechanistic insights are expected from the J1I-MC-GZBU study, in which the effects of retatrutide on mGFR and kidney physiology by multiparametric kidney MRI are investigated. Finally, the combination of a GLP-1 receptor agonist and amylin agonism by cagrilintide/semaglutide is currently under investigation (Table 1).

The smaller mechanistic trials such as the REMODEL trial (NCT04865770) (52) may be able to identify pathways affected by these novel incretin mimetics, and how these differ from the GLP-1 receptor agonists, thereby providing information regarding the role of the different receptors that are being targeted. The TRIUMPH-Outcomes trial, beyond efficacy on clinical outcomes, will provide data on whether the drugs are safe, including in those with more advanced CKD (54). In addition, the larger trials will shed light on whether potential kidney effects are dependent on the administered dose, as not all patients will likely be uptitrated to the highest dosages of the drugs studied in the trials.

With the advent of several new incretin combination drugs, future research must focus on selecting the right patient for the right incretin combination. Here, the combination with other drugs such as RAAS blockers, SGLT2 inhibitors, and mineralocorticoid receptor agonists, which have also displayed renoprotective effects, needs to be taken into account as well. As incretin therapies are relatively costly, cost-effectiveness studies will need to demonstrate in which high-risk population the use of incretins should be prioritized, especially in those without diabetes, where data remain scant.

To summarize, while it is already clear that GLP-1–based therapies will be an integral part of the treatment of DKD owing to their metabolic, cardiovascular, and kidney effects, exciting times lie ahead, with the ongoing development of compounds that will have potentially greater effects on these outcomes, due to more pronounced effects on body weight and glycemia. Furthermore, the extension of these drugs to CKD-affected populations without diabetes and perhaps without obesity has the ability to improve kidney disease prognosis worldwide, as the mechanisms by which these drugs improve function may be driven only in part by normalization of glycemia and body weight.

## Figures and Tables

**Table 1:**
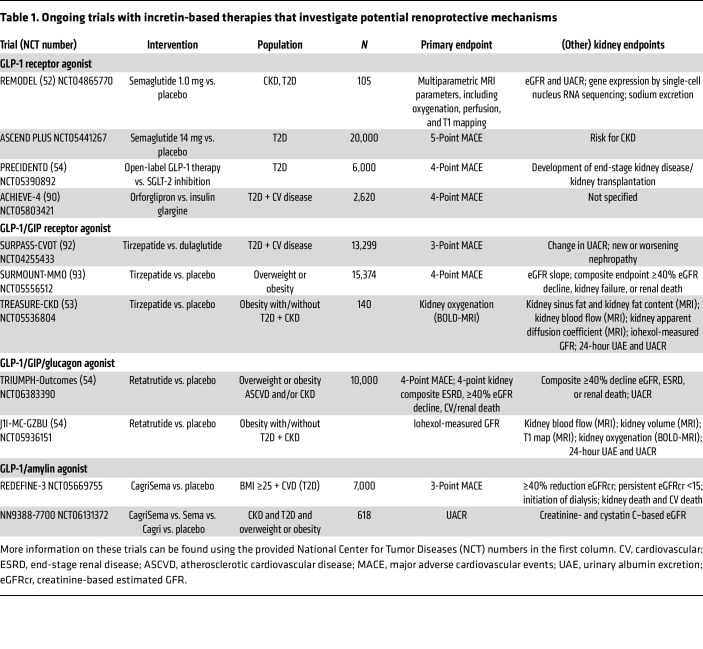
Ongoing trials with incretin-based therapies that investigate potential renoprotective mechanisms

## Version 1 11/03/2025

Electronic publication
